# Epidemiological, clinical and outcomes of pleural tuberculosis: insights from a 646-patient cohort in the province of Khémisset, Morocco

**DOI:** 10.11604/pamj.2025.52.53.48628

**Published:** 2025-10-01

**Authors:** Ismail Ouhammou, Hinde Hami, Arabi Rida, Mezouara Mounia, Ouassima Erefai, Driss Hmouni

**Affiliations:** 1 Laboratory of Natural Resources and Sustainable Development, Faculty of Sciences, Ibn Tofail University, Kenitra, Morocco; 2 Laboratory of Biology and Health, Faculty of Science, Ibn Tofail University, Kenitra, Morocco; 3 Higher Institute of Nursing Professions and Health Techniques, Rabat, Morocco

**Keywords:** Pleural tuberculosis, epidemiology, Khémisset, Morocco

## Abstract

**Introduction:**

pleural tuberculosis, the second most common extrapulmonary manifestation after lymph node involvement, poses a considerable diagnostic and therapeutic challenge. This study examines the epidemiological, clinical, biological, and therapeutic features of pleural tuberculosis cases in Khémisset, a province in Morocco‘s Rabat-Sale-Kenitra region, which reports the country‘s second-highest tuberculosis incidence.

**Methods:**

this retrospective study examined the medical records of patients diagnosed with pleural tuberculosis at the Khémisset Diagnostic Center for Tuberculosis and Respiratory Diseases between 2016 and 2020. A multivariate logistic regression analysis was performed to identify the determinants associated with treatment failure.

**Results:**

over the five-year study period, 646 cases of pleural tuberculosis were recorded, representing 19.8% and 48.8% of all tuberculosis and extrapulmonary tuberculosis cases in our cohort, respectively. The mean patient age was 42 years (standard deviation 22.2), with a male predominance (59.4%, male-to-female ratio of 1.46). A bimodal age distribution was observed, peaking in young adults (20-44 years, 39.6%) and older individuals (>55 years, 34.6%). Geographically, cases were nearly evenly distributed between urban (49.7%) and rural (50.3%) areas. The majority (95.2%) were new pleural tuberculosis cases, while 4.2% had concurrent extrapulmonary or pulmonary tuberculosis involvement. Notably, HIV screening was performed in only 31.1% of cases, all of which yielded negative results. Treatment outcomes were favourable, with a therapeutic success rate of 84.4% and a mortality rate of 8%. Multivariate logistic regression analysis revealed the presence of an association of clinical forms significantly linked to an increased risk of failure. The adjusted model indicates an OR of 2.45 (95% CI: 1.02 - 5.88; P = 0.045).

**Conclusion:**

pleural tuberculosis accounts for 1 in 5 tuberculosis cases locally. Our study reveals that, despite high treatment success rates, a significant proportion of patients with pleural tuberculosis fail treatment, particularly those with mixed symptoms, especially if they have a combination of other forms of tuberculosis, which increases the risk of failure.

## Introduction

Tuberculosis (TB) remains a major global health concern, ranking as the second leading cause of death from an infectious disease worldwide [1]. In Morocco, despite ongoing public health efforts, TB continues to pose a significant challenge, with extrapulmonary tuberculosis (EPTB) representing a considerable proportion of cases [2]. EPTB is defined as an active infection caused by *Mycobacterium tuberculosis* outside the pulmonary parenchyma, capable of affecting multiple organs and tissues [3]. The proportion of EPTB among all TB cases in Morocco has risen steadily, increasing from 28% in 1990 to nearly half (48%) of all cases by 2019. Lymph node and pleural tuberculosis (PTB) are the most prevalent forms, accounting for nearly 70% of EPTB cases [4].

Pleural tuberculosis (PTB), resulting from the infection of the pleural space by *Mycobacterium tuberculosis*, presents diagnostic and therapeutic challenges due to its nonspecific clinical manifestations [5]. This form of TB highlights the need for improved diagnostic and management strategies [6]. PTB may manifest after pulmonary TB, as the bacterial infection disseminates from the lungs to the pleural membranes [7]. This dissemination may occur either concurrently with active pulmonary disease or following reactivation of latent infection, suggesting that the TB infection was preexisting but had remained quiescent for a certain duration [8]. The pathogenesis often involves a hypersensitivity reaction to mycobacterial antigens, triggering pleural inflammation and effusion [9]. The prevalence of PTB effusion exhibits significant geographical variation, reflecting distinct regional epidemiological patterns. In Spain, PTB constitutes 14.3-19.3% of all TB cases, with an incidence rate of 4.8 per 100,000 population [10]. South African data report that accounts for 42% of national cases, with pleural involvement present in 30% of these EPTB presentations [11]. Globally, approximately 41.3% of EPTB involves pleural infection [12]. In Morocco, PTB comprises 30-45% of EPTB cases, as observed in regional studies conducted in Larache (North of Morocco) [13].

Clinical patient outcomes of PTB are strongly associated with socio-economic factors, treatment adherence rate, healthcare system accessibility, and comorbid conditions [14]. A comprehensive understanding of these prognostic factors is essential for developing targeted interventions to improve clinical management and reduce the disease burden. PTB is one of the most common forms of ETB and carries a risk of unfavourable progression. This study aims to analyse PTB epidemiology in Khémisset, Morocco, assessing prevalence, risk factors, clinical patterns, and treatment outcomes. The results will optimise local diagnostic approaches and guide context-appropriate control measures for this significant EPTB manifestation.

## Methods

**Study design:** this retrospective cohort study analysed 646 consecutive cases of laboratory-confirmed PTB diagnosed at the Khémisset Center for Diagnosis of Tuberculosis and Respiratory Diseases during five years (January 2016-December 2020). The analysis utilised complete medical records from the center‘s archives, including socio-demographic characteristics, clinical examination findings, microbiological and histopathological reports, and treatment outcomes.

**Study site:** this investigation was conducted at the Khémisset Diagnostic Center for Tuberculosis and Respiratory Diseases, the principal reference facility for TB screening, diagnosis, and treatment management in Khémisset Province, Morocco. Geographically, this city is situated in northwestern Morocco. It shares borders with Kenitra Province (north), Rabat (west), Meknes Province (east) and Khouribga and Khenifra Provinces (south). From an epidemiological perspective, Khémisset, a predominantly rural province in Morocco‘s Rabat-Salé-Kénitra region, stands out as a critical area for tuberculosis control. With an incidence rate of 110 cases per 100,000 inhabitants in 2022, this rate is higher than the national rate (94 cases per 100,000 inhabitants), underscoring persistent healthcare access challenges and socio-economic risk factors.

### Study population

**Inclusion and exclusion criteria:** we included all laboratory-confirmed and clinically diagnosed cases of pleural tuberculosis during the study period (2016-2020). Cases with incomplete medical records or indeterminate TB diagnoses were excluded to ensure data quality.

**Data collection:** the data collected from patient records and files were systematically recorded in an Excel 365 file. While respecting anonymity, these data, related to the variables of our study, were encoded.

**Variables analysed:** the study evaluated four categories of variables:

***Demographic characteristics:*** age, sex, and residence (urban/rural).

***Clinical parameters:*** case classification (new case, relapse), HIV serostatus (tested/untested, positive/negative), concurrent TB involvement (pulmonary/other extrapulmonary).

***Diagnostic and laboratory analysis:*** histopathological findings (*granulomas* and caseous necrosis in pleural biopsies), pleural fluid macroscopic examination (color, turbidity, and Rivalta test), and pleural fluid microscopic characteristics (protein levels, leukocyte counts, lymphocyte percentage, and adenosine deaminase (ADA) activity).

***Treatment outcomes:*** therapeutic success, failures, loss of sight, and death.

**Diagnostic methods:** diagnosis of pleural tuberculosis requires a comprehensive approach combining clinical evaluation with multiple diagnostic tests. At our center, this process adheres to international guidelines and standards, following these key steps:

***Radiographic imaging (standard chest X-ray):*** typically reveals pleural effusion as the initial finding.

***Pleural and chest wall biopsy:*** histopathological analysis of tissue samples for definitive diagnosis.

***Pleural fluid analysis:*** including macroscopic assessment, cytology and biochemical testing, and adenosine deaminase (ADA) assay, which is a critical immunological marker and early screening tool for tuberculous pleurisy [6]. Direct examination by Ziehl‘s method for *Mycobacterium tuberculosis* is rarely used due to its low sensitivity in PTB diagnosis [6].

**Statistical analysis:** the encoded data were analysed using SPSS version 26 software. Descriptive statistics were computed for all variables, quantitative variables were summarised as ± standard deviations and qualitative variables were expressed as frequencies and percentages. Results were organised in comprehensive frequency distribution tables for clear presentation. Then, a univariate and multivariate binary logistic regression analysis was performed to evaluate and identify the determining factors related to treatment failure. The results are presented as odds ratios (OR) with 95% confidence intervals. Variables with p< 0.05 were considered statistically significant.

**Ethical considerations:** the fundamental principles set out in the Declaration of Helsinki have been respected by obtaining all necessary authorisations and guaranteeing the confidentiality and anonymity of the data collected.

## Results

**Figure 1:**
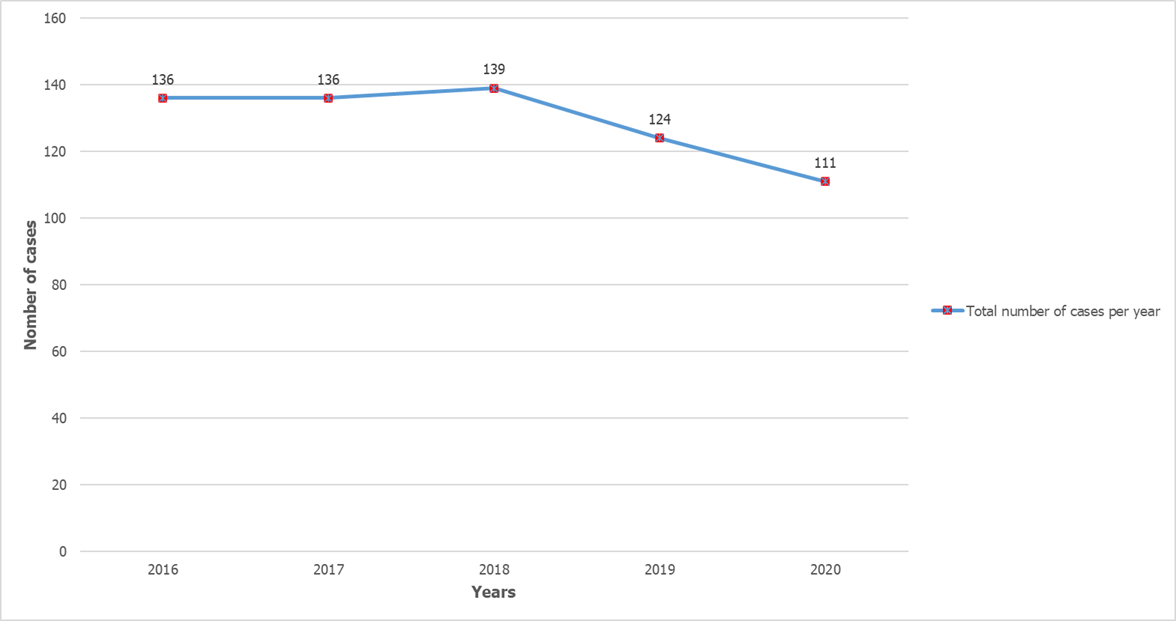
number of cases per year

**Figure 2:**
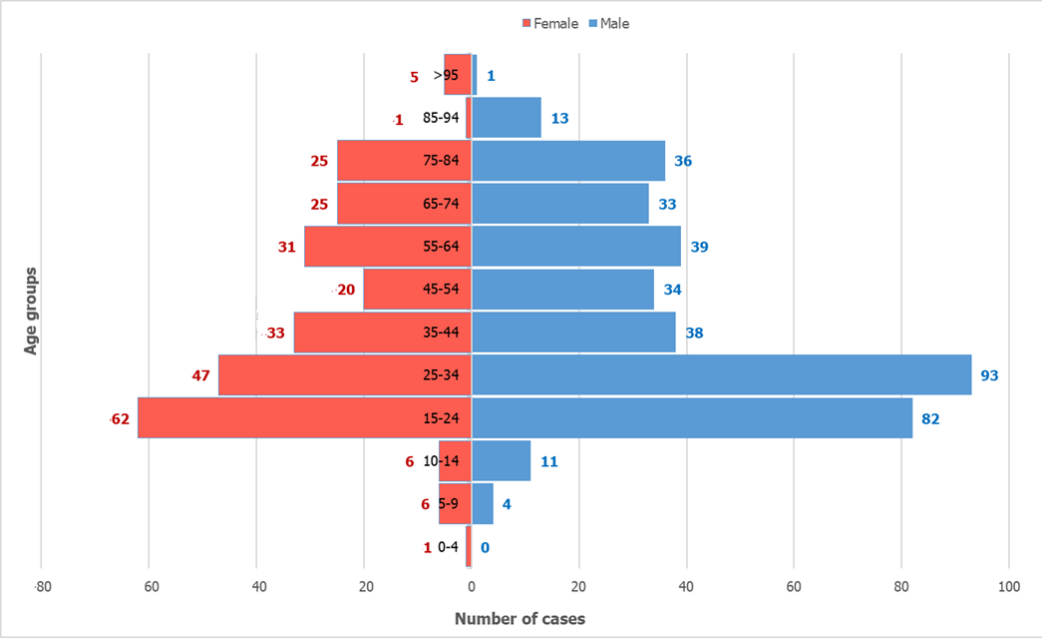
number of cases by age groups and sex

**Epidemiological trends and demographic characteristics:** during our five-year study, we identified 646 PTB cases, representing 19.8% of TB cases (n=3251) and 48.8% of EPTB cases (n=1,325). PTB cases exhibited modest fluctuations between 2016 and 2020, with relative stability in the early years followed by a gradual decline, peaking in 2018 (21.5%) and reaching their lowest point in 2020 (17.2%) (Figure 1). The study population (n=646) demonstrated a mean age of 42 years (standard deviation 22.2) with a male predominance (59.4%, sex ratio 1.46). The bimodal age distribution peaked in young adults (20-44 years; 39.6%) and older adults (>55 years; 34.6%) (Figure 2). Geographic distribution showed near-equivalent representation between urban (49.7%) and rural (50.3%) residences.

**Clinical presentation and diagnostic features of PTB:** the cohort consisted primarily of new TB diagnoses (95.2%, n=615), with relapse cases (2.2%), treatment withdrawals (1.5%), and transferred patients (1.1%) comprising the remaining cases. Among PTB cases, 4.2% (27/646) presented concurrent TB manifestations at other sites. Pericardial (26%), peritoneal (22%), and pulmonary (22%) involvement predominated among these disseminated cases (Table 1 for complete localisation patterns). HIV testing, performed in 201 cases (31.1% of the cohort), showed no positive results. Histopathological analysis revealed that 33% of cases showed normal striated muscle architecture. The pleural fibrous tissue demonstrated characteristic features of TB, including polymorphic inflammatory infiltrates and epithelioid giant-cell granulomas, with frequent caseous necrosis. No evidence of malignancy or other pathogenic organisms was observed. These results strongly indicated caseo-follicular PTB, marked by a specific immune response to *Mycobacterium tuberculosis.* Macroscopic analysis of pleural fluid revealed a cloudy appearance in 88.2% of cases (570/646), with a predominant yellow colouration (69.8%). The Rivalta test was uniformly positive in all cases (100%), confirming the exudative nature of the effusion through elevated protein content (Table 2).

**Table 1:** concurrent TB localization in PTB cases

	Effective	Frequency
**Association with other forms**		
No	619	95.8 %
Yes	27	4.2 %
**Localization**		
Pericarditis	7	26%
Pulmonary	6	22%
Ascites	6	22%
Intestinal	3	11%
Lymph node	2	7%
Neuromeningeal	1	4%
Miliary	1	4%
Osteoarticular	1	4%

**Table 2:** examination of pleural fluid

**Macroscopic examination**				
	**N**	**Missing**	**Frequency**	
**Aspect**	**281**	**165**		
Slightly hematic	15		3.9%	
Slightly turbid	336		88.2%	
Turbid	30		7.9%	
**Color**	**258**	**288**		
Yellow	180		69.8%	
Lemon yellow	36		14.0%	
Orange yellow	42		16.3%	
**Rivalta reaction**	**382**	**164**		
Positive	382		100.0%	
**Microscopic examination**				
	**N**	**Missing**	**Average**	**Standard deviation**
**Cytochemistry**				
Protein dosage (g/l)	384	162	52.77	7.82
White blood cells (/mm3)	382	164	2094.26	2035.52
Red blood cells (/mm3)	382	164	2655.99	6159.28
**Methylene blue staining**				
Lymphocytes (%)	366	180	80.55	12.61
Polynuclear neutrophils (%)	367	179	17.32	13.68
Other	206	340	3.82	2.34
**Immunochemistry**				
Adenosine Deaminase (ADA) (U/L)	255	291	56.85	22.70

Pleural fluid analysis encompassed cytochemical, cytological, and biochemical characterisation (Table 2). Cytochemical evaluation demonstrated elevated protein levels (52.77 g/L, standard deviation 7.82) and marked leukocytosis (2.094 cells/mm, standard deviation 2.036, n=382). Cytological examinations via methylene blue staining revealed lymphocyte-predominant effusions (80.6%, standard deviation 12.6). Immunochemical analysis showed significantly elevated adenosine deaminase (ADA) activity (56.9 U/L, standard deviation 22.7), consistent with tuberculous pleuritis.

**Figure 3:**
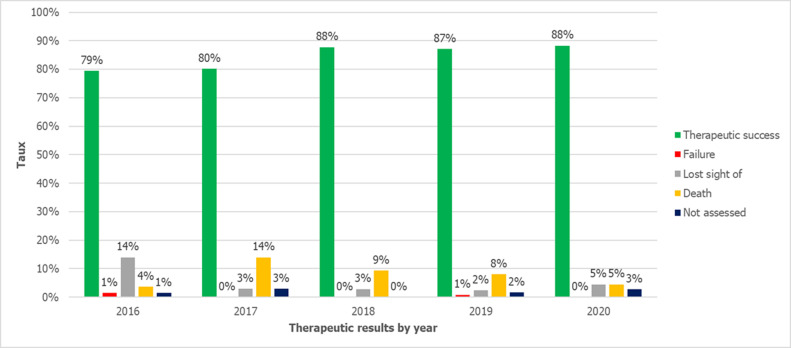
therapeutic outcomes by years between 2016 and 2020

**Treatment outcomes:** our cohort demonstrated significant variability in therapeutic results, with 545 patients (84.4%) completing treatment. However, 52 patients (8%) died during treatment, while treatment failure was rare, occurring in only three cases (0.5%). Challenges in follow-up were evident, with 35 patients (5.4%) lost to follow-up and 11 (1.7%) having unevaluable outcomes, including 7 transferred cases (1.1%) with undocumented final status. A detailed analysis of treatment outcomes by year revealed a notable improvement in success rates, which increased from 79% in 2016 to 88% in 2018, remaining stable at this level until 2020. In contrast, the case fatality rate exhibited fluctuations: it rose sharply from 4% in 2016 to 14% in 2017, then declined to 3% in 2020. The failure rate remained low throughout the study period, recorded at 1% in 2017 and 2019, and 0% in all other years (Figure 3).

Univariate analysis revealed a significant association between forms associated with other forms of TB and treatment failure (unadjusted OR of 2.40; 95% CI: 1.01- 5.65; P = 0.048). No other variables showed a significant association (P >0.05). Logistic regression analysis revealed that age 60 years and over was associated with a reduced likelihood of treatment failure in the adjusted model, although this association was not statistically significant (adjusted odds ratio (OR): 0.61; 95% confidence interval (CI): 0.36-1.03; P = 0.065). Neither male gender nor urban residence showed a significant association with treatment failure, nor did a history of tuberculosis. Conversely, the presence of a combination of clinical forms was significantly associated with an increased risk of failure (adjusted OR: 2.45; 95% CI: 1.02-5.88; P = 0.045), while ADA testing showed no significant association with treatment outcome (adjusted OR: 1.10; 95% CI: 0.65-1.87; P = 0.730) (Table 3).

**Table 3:** analysis of factors associated with treatment failure (univariate and multivariate regression models)

Variable	Unadjusted OR (95% CI)	P -value	Adjusted OR (95% CI)	P-value
Age group ≥ 60 years old	0,58 (0,34–1,00)	0,052	0,61 (0,36-1,03)	0,065
Male gender	0,91 (0,59-1,40)	0,678	0,89 (0,56-1,38)	0,600
Urban environment	0,69 (0,44-1,09)	0,112	0,68 (0,42-1,10)	0,111
History of TB	1,12 (0,45-2,78)	0,800	1,08 (0,42-2,76)	0,870
Associated forms	2,40 (1,01-5,65)	0,048	2,45 (1,02-5,88)	0,045
High ADA	1,05 (0,63-1,76)	0,854	1,10 (0,65-1,87)	0,730

## Discussion

This study provides an overview of the epidemiological situation of PTB in the province of Khémisset, Morocco, revealing that it represents nearly 20% of all TB cases and almost half of EPTB instances. These findings demonstrate the significant burden of this form of TB in this region. PTB is a major extrapulmonary manifestation of TB, with considerable epidemiological variation across regions. Globally, it accounts for approximately 5% of all TB cases, ranking as the second most common extrapulmonary form after tuberculous lymphadenitis [15,16]. However, prevalence rates differ significantly by region, with around 4% of cases in the United States [15], while in Nigeria, it predominates, representing 67% of EPTB cases [17]. This geographical disparity is further evident in the disease burden, affecting up to 30% in high-burden TB regions [18]. Our study revealed that PTB affects a broad range of age groups, with particularly high prevalence among both young and elderly populations. This finding aligns with global epidemiological variations. Brazil reports a significant burden among children and adolescents [19], while studies in Yaoundé (Cameroon) and Madagascar found a median age of 40 years and 46.60 years, respectively [9]. Similarly, Tanzanian data revealed a lower mean age of 33 years [20].

Demographic analysis of PTB cases reveals a significant prevalence of male patients, a trend consistently observed across multiple studies. In Marrakech, Morocco, 58.3% of affected individuals were male [5]. Similarly, in Yaoundé, males accounted for 54% of 200 cases [14]. Tunisian data further corroborated this disparity, with 60.4% being male [21]. Studies in Tanzania, Brazil, and Madagascar reported even higher male prevalence rates of 56.6%, 62.7%, and 61.08%, respectively [19], [20]. This persistent gender disparity suggests potential biological, behavioural, or socio-economic factors influencing susceptibility concerns [9, 19, 20].

PTB can occur alongside pulmonary TB or due to reactivation of latent infection [16, 22, 23]. Analysing case distribution by setting, year, and clinical category provides key epidemiological and clinical insights [24]. This study highlights the association between PTB and other forms of TB, emphasising its polymorphic manifestations [25]. Such clinical complexity complicates diagnosis and management, often requiring multifaceted treatments and close medical monitoring for optimal outcomes [21]. The diagnosis of PTB requires a systematic approach combining clinical evaluation, imaging (radiography, ultrasound, CT, MRI), and laboratory analysis [26]. Chest radiography remains the primary diagnostic tool post-symptom onset [27], but accurate diagnosis depends on integrating radiological, clinical, and biomarker findings [28]. Pleural fluid analysis is critical, with adenosine deaminase (ADA) serving as a key biomarker despite its limited specificity due to cross-reactivity with other conditions [29,30]. Diagnostic precision is enhanced by a high lymphocyte-to-neutrophil ratio, which achieves over 90% accuracy [31]. Critical ADA thresholds include 40 IU/L (100% sensitivity, 79% accuracy) [32], 95 IU/L (100% sensitivity, 75% accuracy) [33], and <35 IU/L (85.7% sensitivity) [34], with median levels in TB patients reaching 70 IU/L [35].

Normal pleural fluid typically appears as a clear, straw-colored transudate with low protein content (<2.5 g/dl), whereas tuberculous pleural effusions exhibit exudative characteristics, including elevated biomarkers such as adenosine deaminase (ADA) (>35 IU/L) and a high LDH/ADA ratio, demonstrating 93.9% sensitivity and 87% specificity for diagnosis [18,36]. In Brazil, histopathological confirmation was achieved in 75% of cases [19], with tuberculous pleurisy accounting for 70.28% of effusions [31]. Pleural biopsy remains the most reliable diagnostic method, emphasising its critical role in accurate diagnosis. Additionally, comorbidities like diabetes [7,37] and cardiovascular disease [9,38] are prevalent in pleural TB patients, highlighting the need for comprehensive management to improve outcomes [21].

The complex relationship between HIV and TB is particularly pronounced in pleural TB, necessitating a comprehensive management approach that includes systematic HIV screening [39]. Indeed, people living with HIV face a 19-fold higher risk of developing active TB compared to the general population [40], underscoring the critical need to integrate TB screening and preventive therapy into HIV programs. However, in our study, only 31.1% of patients underwent HIV testing, a gap that may reflect missed opportunities for dual diagnosis and optimised care.

Our study achieved a success rate of 84.4%, in line with results obtained in other regions where tuberculosis is endemic. In Madagascar, a comparable cohort achieved a success rate of 84.6% (HIV-negative patients) [41], while studies in Cameroon reported a success rate of 80.7% in HIV-negative groups and 72% in HIV-positive groups [42]. These consistent results in a variety of settings confirm the efficacy of current anti-tuberculosis treatments, even in populations with HIV comorbidity [43]. Despite therapeutic success, our case-fatality rate of 8% deserves attention. Our rate was higher than reports from Cameroon (2.6-4.9%) [42] but lower than the 10.1% from Madagascar [41]. This variation probably reflects differences in disease severity at presentation and in care protocols. The low failure rate in our cohort (0.5%) reinforces the efficacy of treatment, while the 5.4% loss to follow-up and 1.9% non-evaluable outcomes (including 1.1% transfers) highlight systemic shortcomings in patient follow-up [43].

This study shows that treatment failure is associated with a combination of clinical forms, doubling the risk after adjustment. While the confidence interval remains wide, indicating some variability in the data, this association is significant. The other variables, age, gender, place of residence, history of tuberculosis and high ADA levels, are not statistically associated with treatment failure. However, certain trends emerge. For instance, the odds ratios for patients aged 60 and over are less than 1 and close to the significance threshold. The confidence intervals, which indicate the margin of error in the predictions, suggest uncertainty, perhaps due to the sample size or population variability. These factors necessitate caution when interpreting the data, as well as further studies to confirm or refute these results. Other studies have shown that treatment failure in PTB is linked to clinical, biological and social factors. These include drug resistance, comorbidities such as diabetes, malnutrition, advanced age, and radiological characteristics. Multidrug resistance and empyema can also be added to this list [44-47].

This research has many strengths. It is based on information gathered in contexts of high social and economic precariousness, enabling an accurate account to be given of the challenges involved in treating pleural tuberculosis in similar settings. The retrospective review enabled a large number of patients to be included over a long period of time, providing a clear picture of therapeutic approaches and clinical issues. Multivariate analysis identified a factor independently associated with treatment failure, thereby facilitating a more profound comprehension of this particular form of tuberculosis. However, there are important limitations. The study‘s retrospective design makes it vulnerable to selection bias and inconsistent data quality because some clinical information was missing or incomplete. In addition, HIV testing was not performed systematically in the study population, making it difficult to assess its influence on treatment outcomes. While these findings apply to similar settings, they may not generalise to contexts with better diagnostic resources or healthcare organisations.

## Conclusion

The results of our study highlight the importance of rigorous monitoring of patients with pleural tuberculosis. They also emphasise the need for careful follow-up of disease progression, treatment effectiveness, and screening for HIV co-infection to optimise care. Although pleural tuberculosis accounts for 20% of local cases, a significant proportion of patients fail to complete their treatment, particularly those with other forms of tuberculosis, which exacerbates the risk of treatment failure. These results call for increased awareness, early diagnosis, and the establishment of standardised treatment protocols to improve the quality of care.

***What is known about this topic***

- *Pleural tuberculosis accounts for approximately 30% of extrapulmonary tuberculosis cases reported in Morocco;*
- *This form of tuberculosis is also rare in children under the age of 14.*

***What this study adds***

- *This study is the first comprehensive epidemiological analysis of pleural tuberculosis in the province of Khémisset, Morocco;*
- *It reveals notable variation in the prevalence and mortality of this disease over time, while highlighting the impact of socio-demographic factors on its evolution;*
- *The results indicate that age, gender and place of residence are determining factors in the disease, particularly when it is associated with other forms of tuberculosis.*
